# A comprehensive study on the mechanical effects of implant-supported prostheses under multi-directional loading and different occlusal contact points

**DOI:** 10.1186/s12903-023-03016-9

**Published:** 2023-05-29

**Authors:** Sangmyeong Tak, Yuwon Jeong, Jong-Eun Kim, Jee-Hwan Kim, Hyeonjong Lee

**Affiliations:** 1 Doctoral student, Department of Prosthodontics, School of Dentistry, Pusan National University, Yangsan, Republic of Korea; 2 Doctoral Course Student, Department of Prosthodontics, College of Dentistry, Yonsei University, Seoul, South Korea; 3 Professor, Department of Prosthodontics, College of Dentistry, Yonsei University, Seoul, South Korea; 4 Clinical Associate Professor, Department of Prosthodontics, Yonsei University College of Dentistry, 50-1, Yonsei-Ro, Seodaemun-Gu, Seoul, 03722 South Korea

**Keywords:** Dental implant, Implant-supported prosthesis, Vector analysis, Stress, Occlusion

## Abstract

**Aims:**

To evaluate screw loosening and fracture load and angular deviation of a single implant-supported prosthesis under multi-directional loading condition at three different occlusal contact points.

**Methods:**

A total of 40 metal crowns were cemented to external connection implants and were embedded vertically and obliquely. The occlusal surface of the crown was designed with three flat surfaces, contact a, b, and c, representing outer and inner 20-degree inclination for buccal and lingual cusps. The angular deviations of implant crown under static 50N of loading were measured. And screw removal torque was evaluated before and after 57,600 load cycles. Then, fracture load was measured for each specimen. Data analysis was performed using one-way analysis of variance test of significance followed by Tukey honest significant difference (HSD) test(*p* < 0.05).

**Results:**

Angular deviation results showed statistical significance between all contact points in vertically embedded group compared to obliquely embedded group, which showed similar results between contact A and B compared to C. In the other hand, screw loosening evaluation did not show statistical significance among the tested groups. And for the fracture load evaluation the maximum values reached twice the yield values in all contact areas.

**Conclusions:**

Mechanical effects were different regarding to diverse loading direction and contact points. The results of this study suggest that the stress concentration might increase in unfavorable vector direction.

## Introduction

The development of a surgical plan for implant treatment necessitates due consideration of a definitive prosthetic plan, comprising various factors such as prosthesis material, number of restored teeth, aesthetic factors, the condition of the antagonist tooth, available space, anatomy of the edentulous ridge, implant distribution and cantilever length, patient preference and compliance [1]. Such careful consideration ensures the provision of a comprehensive surgical plan that caters to the individual needs of the patient and maximizes the chances of long-term success of the implant treatment.

In addition to the aforementioned factors, excessive occlusal force constitutes a crucial influencing factor that significantly impacts the success of implant treatment. Inadequate distribution of occlusal forces can lead to various mechanical complications, including screw loosening, deformation, and fracture of the implant and abutment, ultimately hampering the efficacy of the implant treatment [2]. The occlusal loading patterns of implants hold particular relevance in determining implant stability and peri-implant stress/strain distribution [3]. Furthermore, the relationship between abutment displacement and micromotion on dental implant is found to vary with respect to the loading direction and implant insertion angle.

The geometric design of the definitive restoration plays a crucial role in determining stress distribution and, thereby, the efficacy of the prosthesis in the long run [4]. Additionally, stress concentration is known to significantly impact bone tissue. While the initial stability of the implant relies on the quantity of cortical and trabecular bone in the vicinity, inadequate distribution of occlusal forces can lead to implant failure despite optimal initial stability [5].

According to a systematic review conducted by Naert et al. [6], it was found that regardless of the length of the implant, the highest stresses were consistently observed at the neck of the implant. This phenomenon is explained by the fact that the prosthesis level, rather than the bone-implant interface, is the most affected area. Additionally, occlusal load is not a unidirectional load as it occurs in various areas of the occlusal surface, depending on the mandibular excursions.

To accurately simulate the clinical condition of occlusal forces, it is important to evaluate loading unsder various directions. Oblique loads are considered more realistic since they mimic mastication movements and have been shown to cause the highest stress in cortical bone surrounding dental implants. Consequently, implant angulation can create unfavorable loading conditions that may result in restoration failure [7].

However, it should be noted that masticatory forces can vary considerably among individuals, and there is currently a lack of studies that comprehensively evaluate stress concentration under various loading directions that reflect real-life masticatory movements. Additionally, individuals who exhibit parafunctional habits such as clenching or grinding can generate excessive masticatory loads that may further complicate the implant treatment and compromise the success of the restoration.

Recent advances in technology have introduced new methods to improve the longevity and success rate of implant-supported restorations [8]. These techniques aim to reduce the risk of mechanical complications that can result from improper loading of the implant. However, it is important to note that further investigation is needed to ensure the safety and effectiveness of these new technologies.

Previous research has primarily examined stress concentration in implant-supported protheses through the consideration of loading direction condiitions such as vertical or oblique forces. However, the stress experienced by the restoration may be influenced by not only the loading direction but also the loading contact point. Thus, the aim of this study is to assess the stress and torque distribution in implant-supported prostheses through the analysis of diverse loading directions and contact points.

It is hypothesized that implant-supported prostheses subjected to multi-directional loading conditions will exhibit higher stress and torque distribution compared to those subjected to single directional loading conditions.

## Materials and methods

**Fig. 1:**
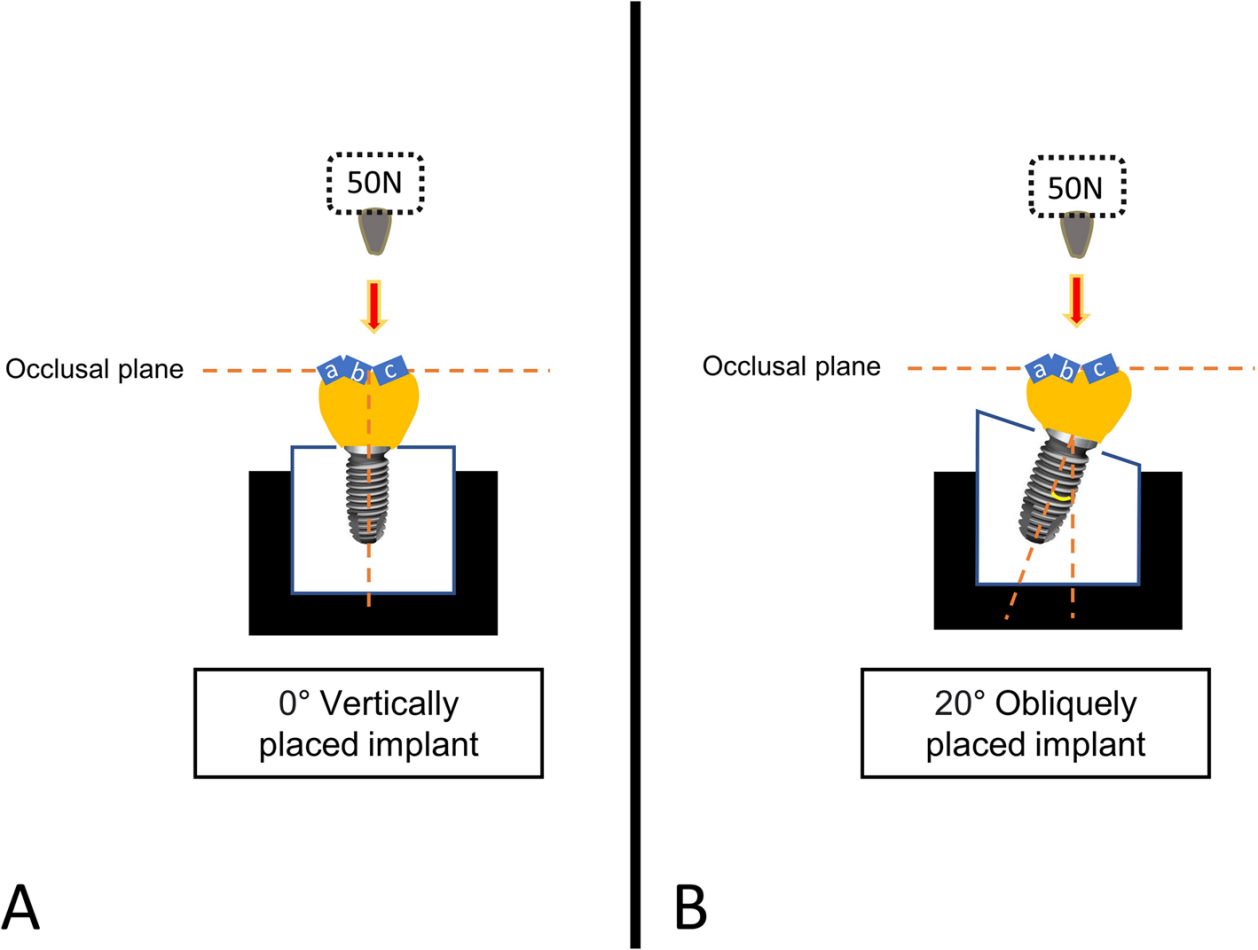
Schematic description of experimental design. **A**. Vertically embedded group. **B**. Obliquely embedded group

This study was designed to evaluate the effects of angular deviation under static loading, screw loosening under cyclic loading, and subsequent fracture loading on the specimens evaluated. The specimens were divided into two groups based on the angle of placement by embedding the specimen vertically and obliquely (Fig 1).

**Fig. 2:**
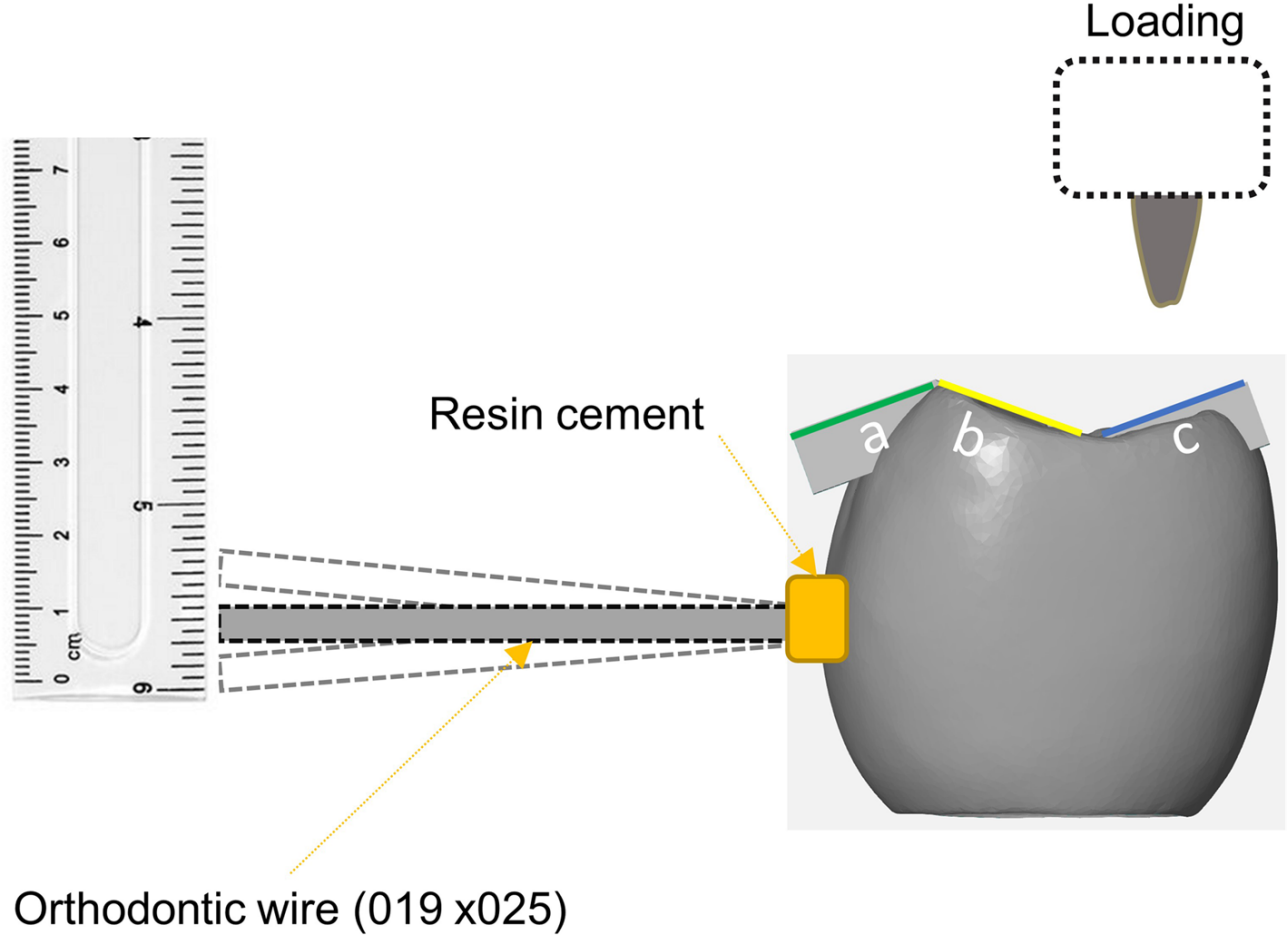
Schematic description of angular deviation evaluation

In order to evaluate angular deviation during static loading, an orthodontic wire measuring 0.19 x 0.25 was bonded onto the buccal surface of the metal crown. Loading was then applied to each contact area to assess the tilting effect at the bottom of the wire (Fig. 2). The assessment of screw loosening was conducted by measuring the removal torque both before and after the loading process. The fracture of each specimen was evaluated using an Universal Testing Machine (UTM) (Instron 3345, Instron Co., Norwood, Massachusetts).

For this study, 40 dental implants of external connection (Dio Implant Co. Ltd., Busan, Republic of Korea) were selected. All fixtures were 10 mm in length, 4.5 mm in diameter and with 0.8 mm screw thread pitch. The abutments were attached to the fixtures with an abutment screw that was torqued to 30 Ncm, as recommended by the manufacturer.

**Fig. 3:**
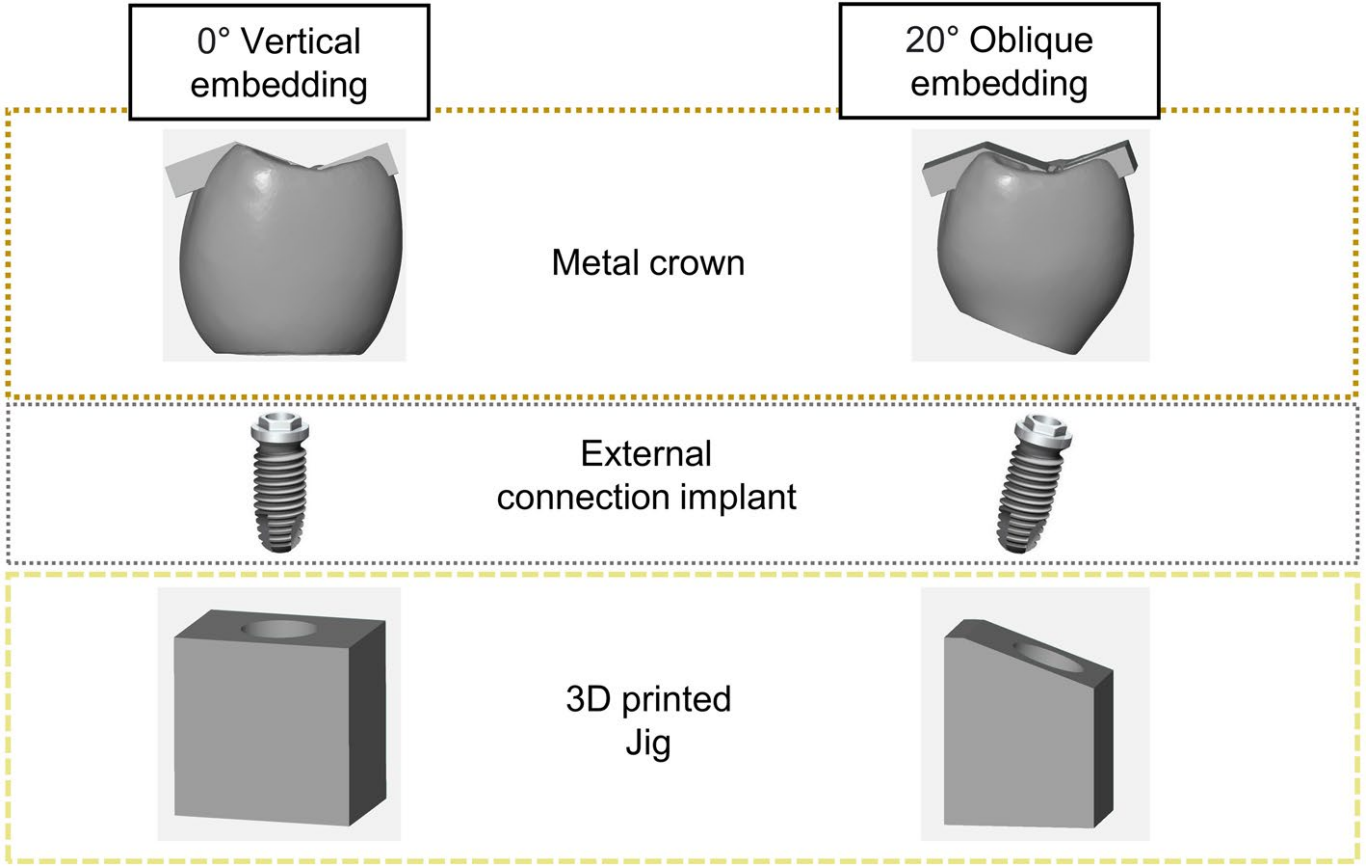
Schematic description of experimental design using flat jig and 20 degrees tilted jig

**Fig. 4:**
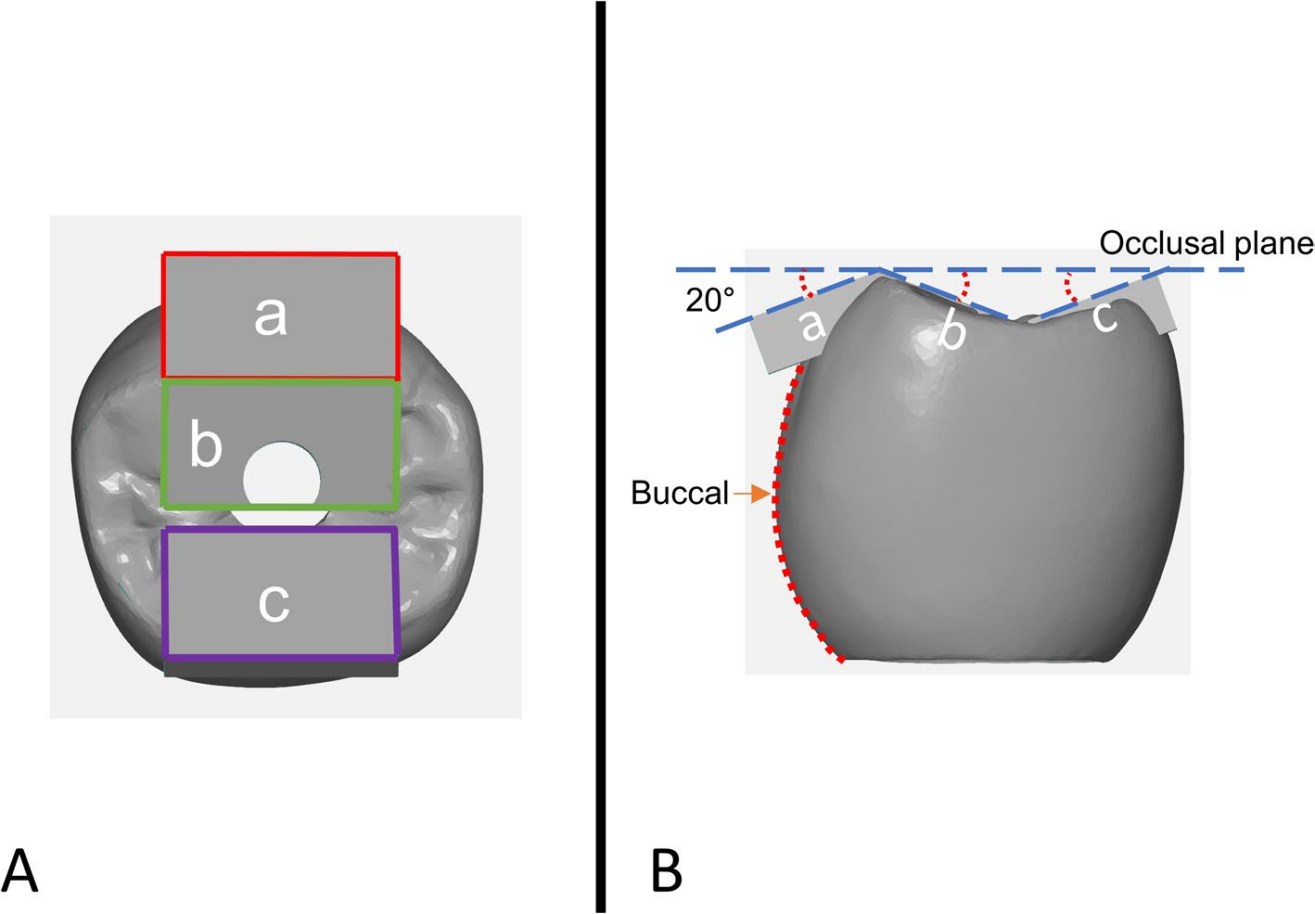
Occlusal contact points. **A**. Contact points division as **A**, **B** and **C**. **B**. Flat surface design with 20 degrees of inclination from occlusal plane

Two rigid resin mounting jigs were custom-designed for this study. The first jig featured a flat surface to embed the specimens vertically, while the second jig had a 20° inclination to embed the specimens obliquely (Fig. 3). To simulate varying occlusal force directions present in actual mastication, the occlusal surface of the crown was modified by adding three rectangular surfaces, designated as contact points A, B, and C (Fig. 4). Twenty implants were embedded to the flat resin block and twenty were embedded to the tilted resin block using autopolymerized acrylic resin. These jigs were fabricated to place specimens into the load frames of the loading machine.

**Fig. 5:**
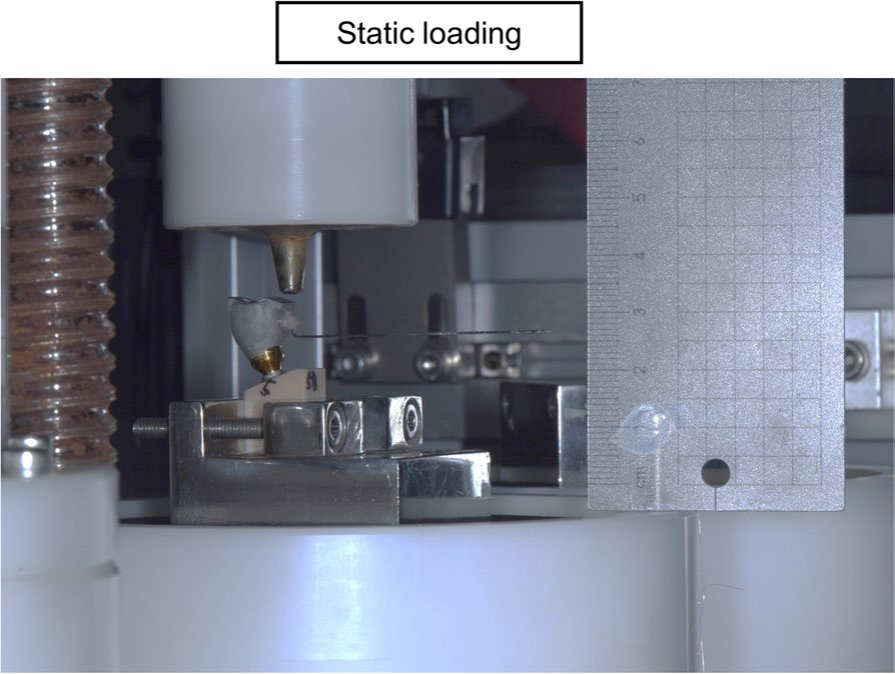
Measurement of angular deviation using a ruler in the apex of orthodontic wire

Subsequently, forty metal crowns were milled and were cemented to the implant abutment using a resin modified glass ionomer cement (GC Fujicem 2, GC, Luzern, Switzerland) using a standardized protocol, excess was removed after cementation. Prior to cementation, the intaglio surface of the metal crowns was subjected to airborne particle abrasion using 50µm Al2O3 at a pressure of 0.15 MPa at a distance of approximately 2 cm. After cementation, angular deviation evaluation was performed measuring the tilting effect at the bottom of the wire(Fig. 5).

**Fig. 6:**
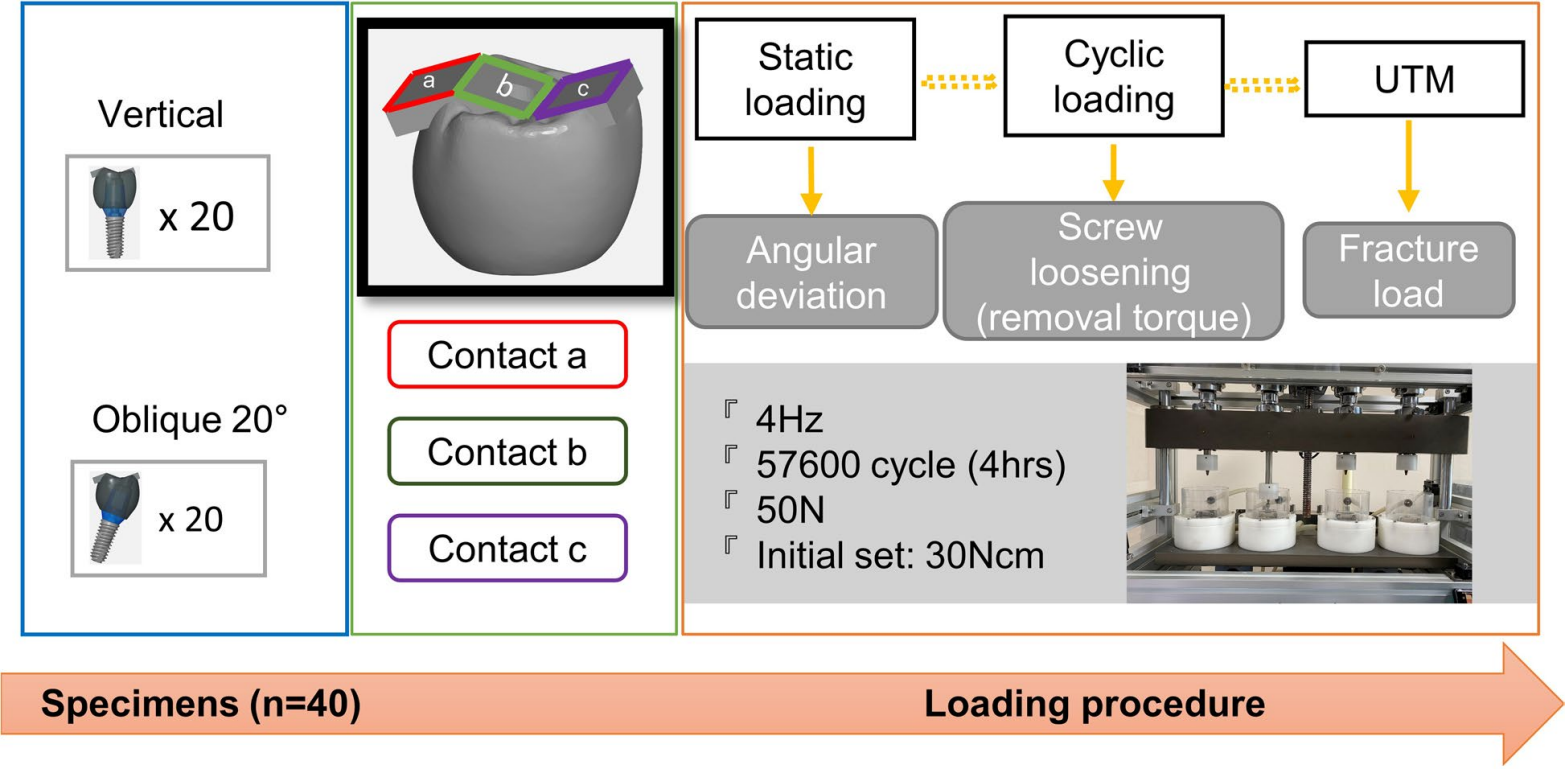
Schematic description of material and methods

After the evaluation of angular deviation, the metal wires were removed, and the specimens were subjected to cyclic loading. The parameters utilized in this study to induce screw loosening were as follows: a frequency of 4Hz, a load of 50N, and a total of 57,600 cycles (approximately equivalent to 3 months of masticatory function), with an initial torque setting of 30Ncm [9–13]. All restorations were positioned centrally in test blocks placed in the machine and the load was applied to each contact surface (Fig. 6).

**Fig. 7:**
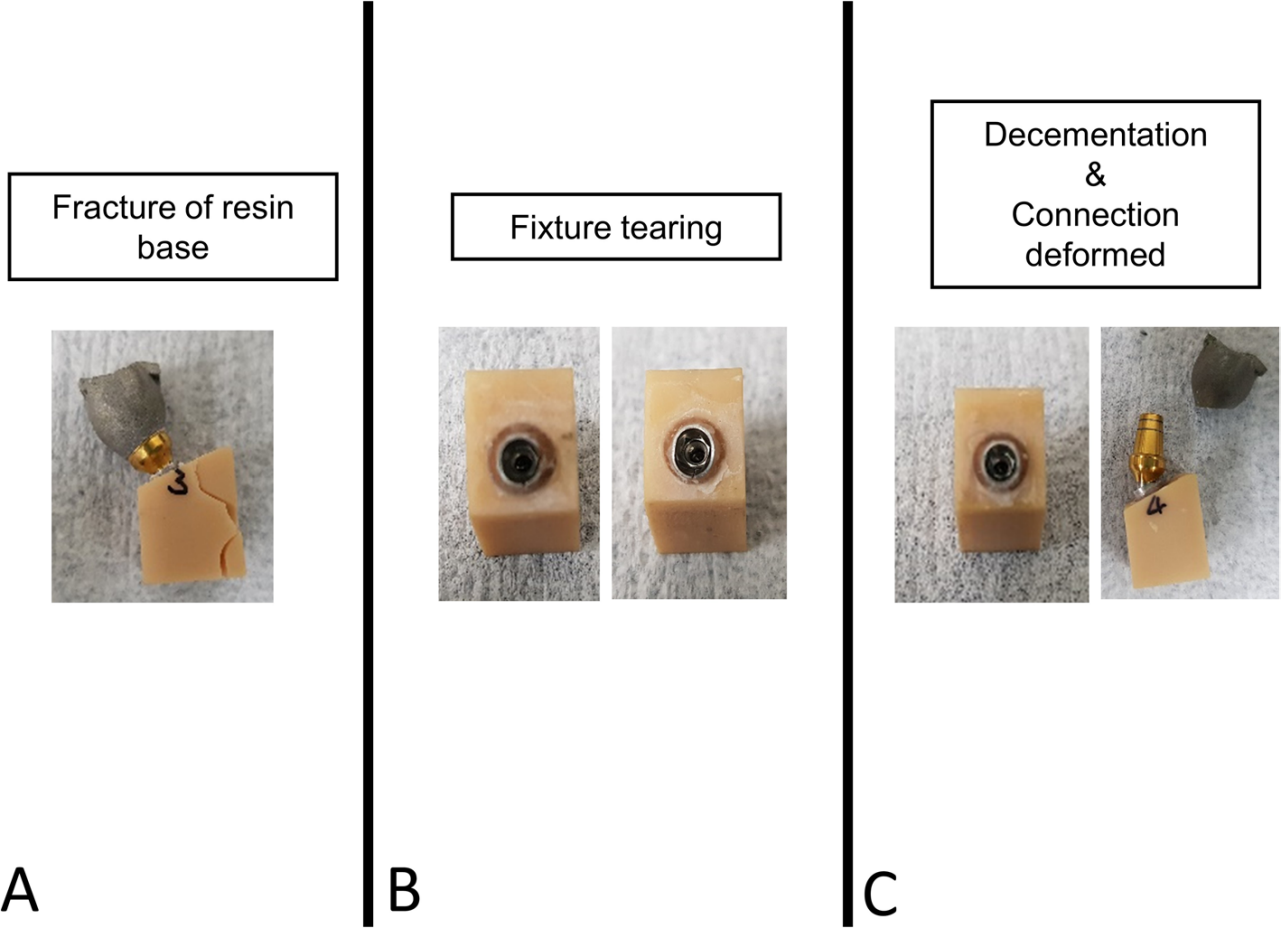
Fracture loaded specimens. **A**. Fracture of resin jig base. **B**. Implant fixture tearing. **C**. Decementation and deformation of connection

**Fig. 8:**
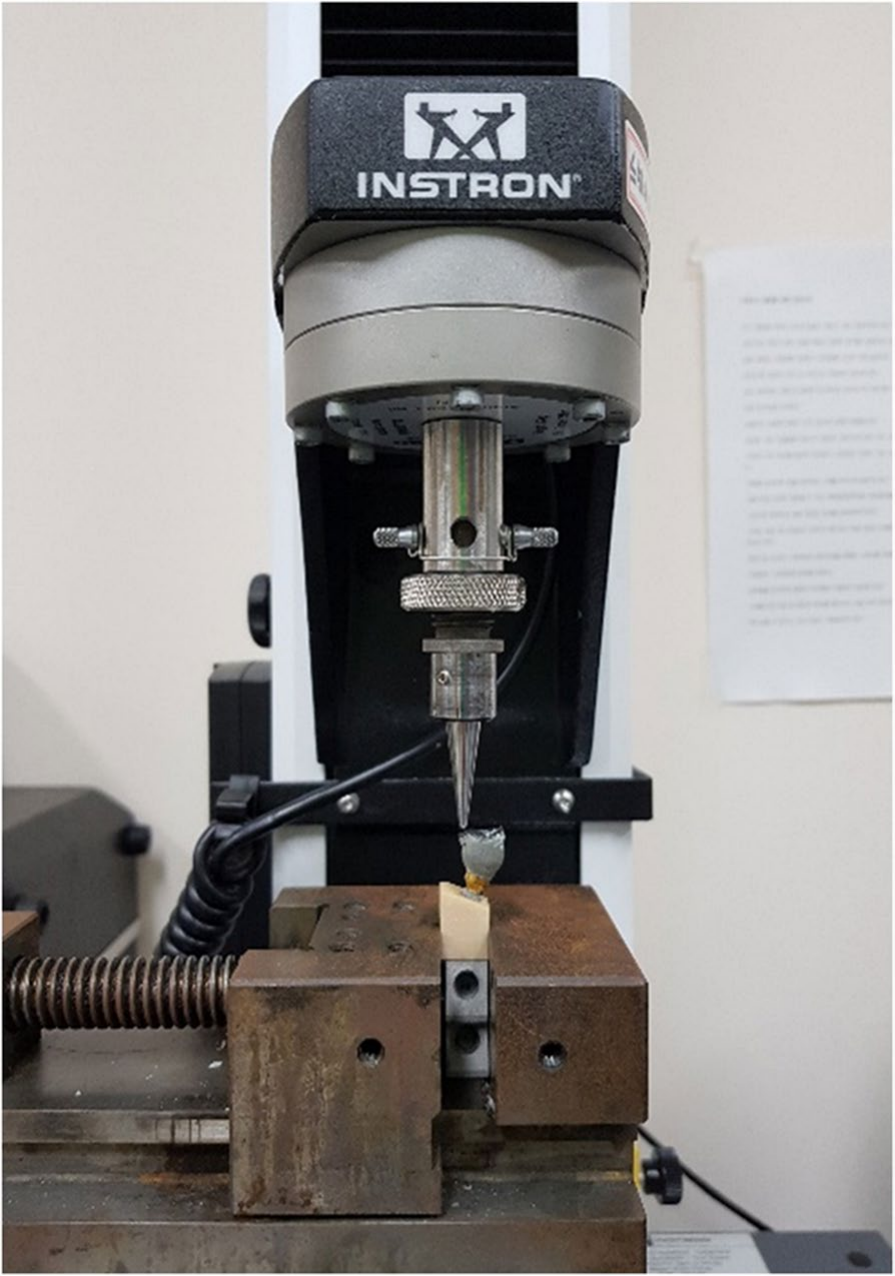
Universal testing machine used in this study (Instron 3345, Instron Co., Norwood, Massachusetts)

Throughout the loading process, some specimens exhibited dislodgement of the metal crown from the implant, resulting in misalignment (Fig. 7). The specimens survived to the loading procedure were loaded until fracture using a UTM with 1mm/min of compressive load at each contact point (Fig. 8). The maximum loading force to fracture values (N) were recorded automatically.

Statistical analysis was performed by conducting a normality test using the Kolmogorov-Smirnov test, followed by one-way ANOVA and post hoc Tukey’s test for multiple comparisons, with a predetermined significance level (α=0.05).

## Results

To evaluate each contact area overall values were evaluated and were divided as follows, V-a (Vertical-contact a), V-b (Vertical-contact b), V-c (Vertical-contact c), O-a (Oblique-contact a), O-b (Oblique-contact b) and O-c (Oblique-contact c). A total of 40 specimens were evaluated, some of them presented decementation of the crown from the implant abutment but all survived to the loading process.

Overall results showed no statistical differences between the specimens embedded vertically and obliquely under loading. This observation can potentially be attributed to the limitations in evaluating internal structural stresses. On the other hand, there were significant differences observed in the loading forces among the contact areas tested.

**Fig. 9:**
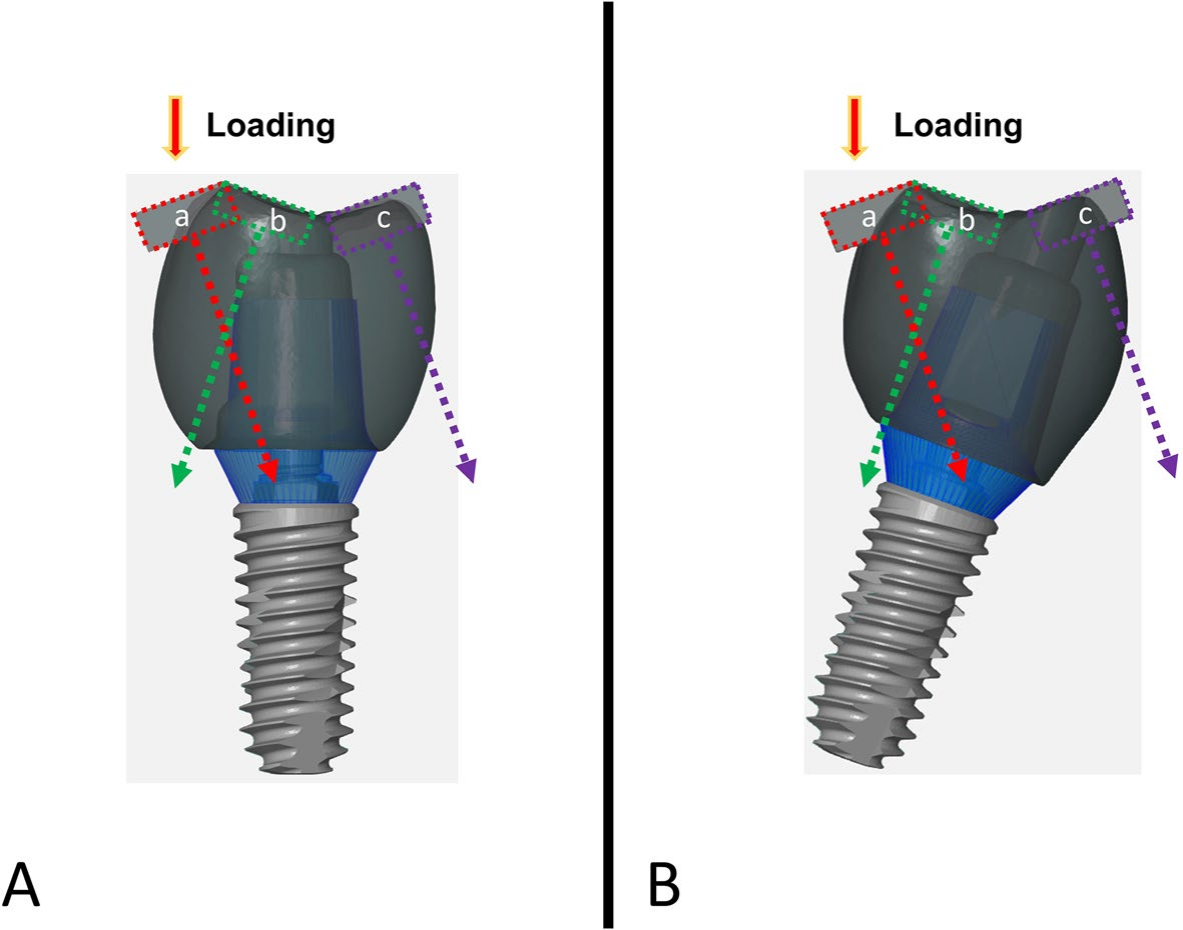
Occlusal vector force regarding to each contact point. **A**. Vertical loading group. **B**. Oblique loading group

**Fig. 10:**
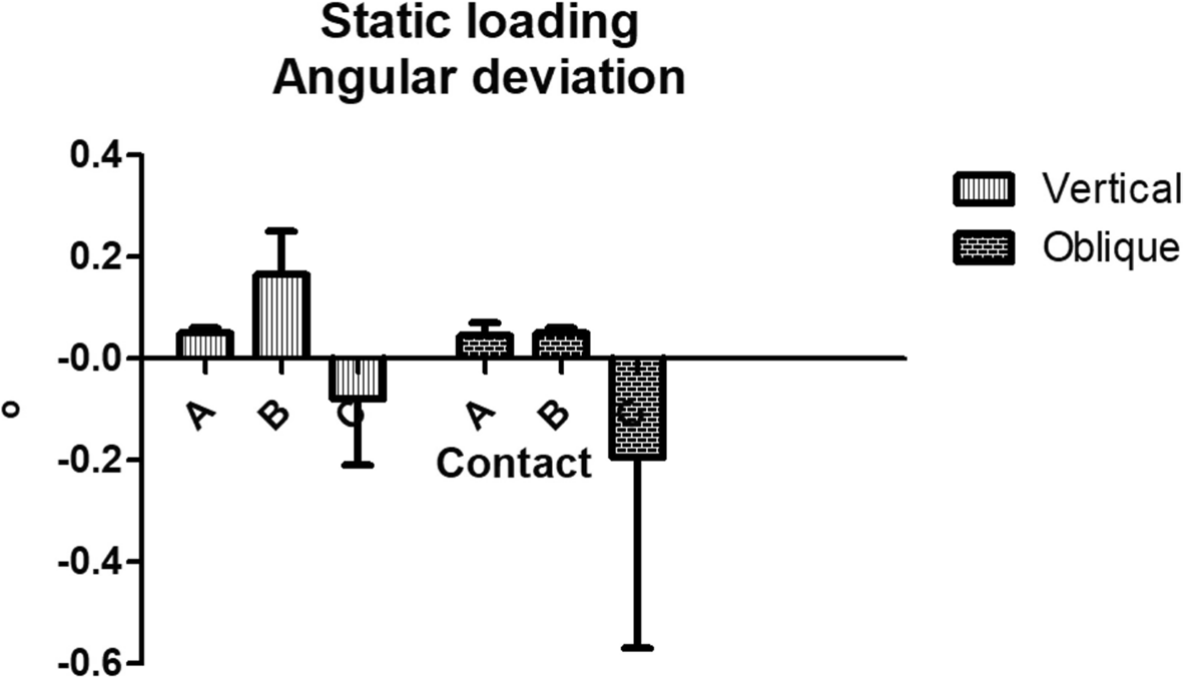
Graph of overall results of angular deviation

The visualization of the occlusal vector regarding to the contact point is shown in Figure 9. Angular evaluation results are shown in Table 1. The angular deviation among the vertically embedded group showed significant differences between all contact area while the obliquely embedded group showed similar results between contact a and b from contact c. (Fig. 10).

**Table 1:** Overall results of angular deviation

	**Mean Angular deviation (o)**	
**Contact point**	**Vertical**	**Oblique**
a	0.06 ± 0.04^Aa^	0.02 ± 0.07^Aa^
b	0.25 ± 0.08^Ab^	0.04 ± 0.06^Ba^
c	-0.21 ± 0.05^Ac^	-0.57 ± 0.18^Bb^

^*^Same upper cases letters in row indicate no statistical significance between the vertically and obliquely embedded groups within the each contact point (a,b and c)

^*^Same lower cases letters in column indicate no statistical significance among different contact points (a, b and c) within the vertically and obliquely embedded groups

**Fig. 11:**
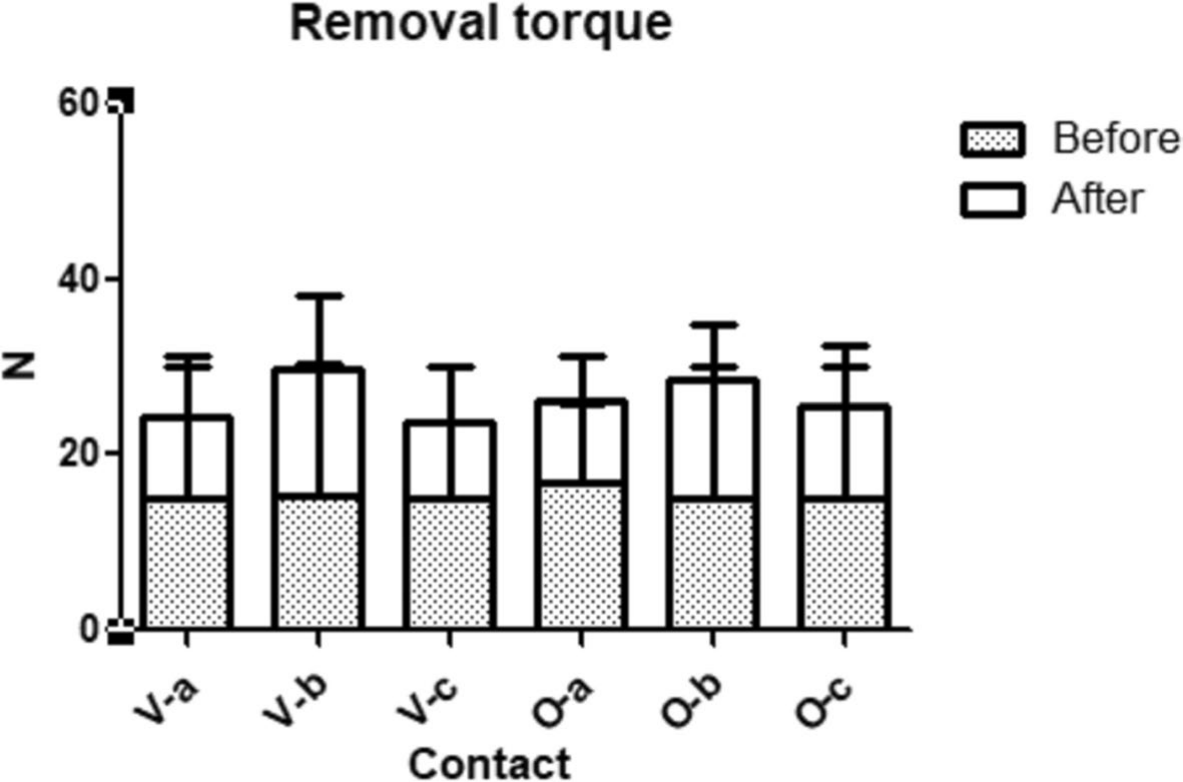
Graph of overall results of torque removal before and after loading among vertical and oblique groups of each contact point

Removal torque of before and after loading was evaluated and are shown in Table 2. The overall results between vertically and obliquely embedded groups did not show significant differences. Though, vertically embedded group showed significant differences after loading procedure between the contact area while obliquely embedded group showed significant differences before and after loading procedure regarding to the contact area (Fig. 11).

**Table 2:** Overall results of removal torque before and after cyclic loading

	**Removal torque (Ncm)**		***P* value (< 0.05)**
	**Before**	**After**	
V-a	30.1 ± 0.1^Aa^	16.2 ± 2.4^Ba^	0.000*
V-b	30.2 ± 0.3^Aa^	22.1 ± 6.0^Bb^	0.000*
V-c	30.1 ± 0.1^Aa^	14.1 ± 2.2^Ba^	0.000*
O-a	25.8 ± 7.8^Aa^	14.3 ± 4.4^Ba^	0.000*
O-b	30.2 ± 0.2^Ab^	19.8 ± 7.0^Bb^	0.000*
O-c	30.1 ± 0.1^Ab^	17.3 ± 3.4^Bab^	0.000*

^*^Same upper cases letters within row indicate no statistical significance between before and after loading

^*^Same lower cases letters in column indicate no statistical significance among different contact points (a, b and c) within the vertically and obliquely embedded groups

**Fig. 12:**
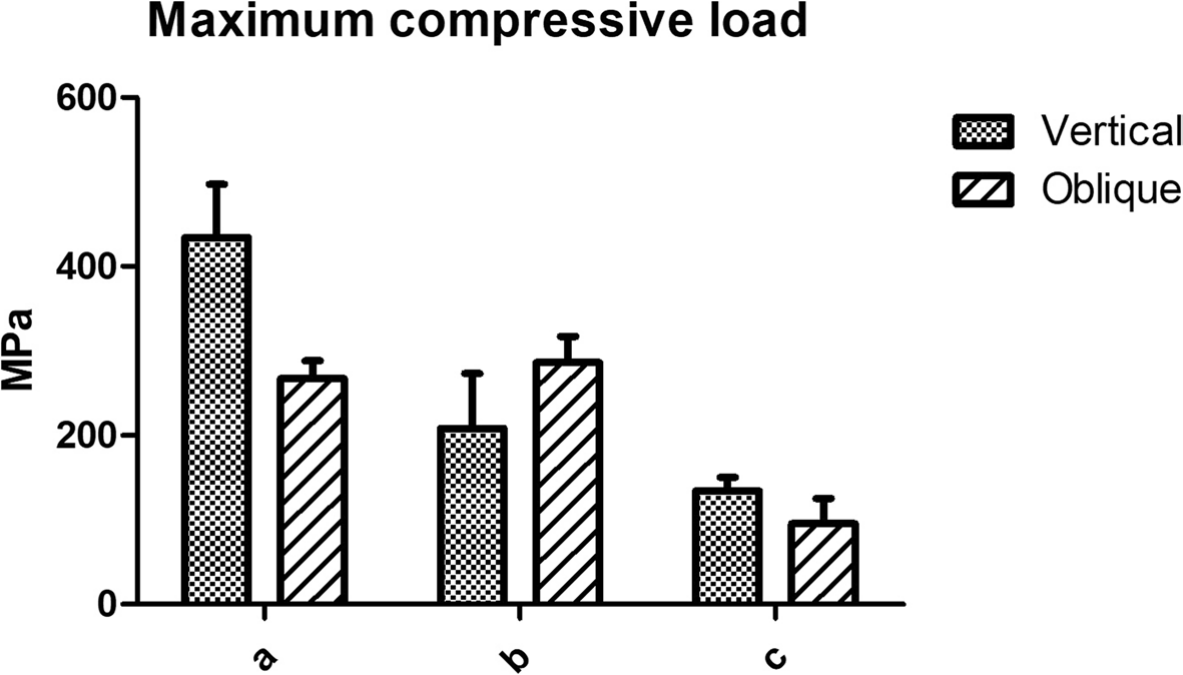
Graph of overall results of compressive strength between vertical and oblique load of each contact point

In addition, fracture evaluation was performed using a UTM and overall results are shown in Table 3. At the maximum compressive load, contact a showed similar values before and after loading procedure while the other contacts were significantly different. In the other hand, compressive strength was significantly different between yield and maximum values (Fig. 12). Indeed, the maximum values reached almost twice the yield values in all contact area. While no significant difference was found within each vertically and obliquely embedded groups.

**Table 3:** Overall results of compressive strength

**Contact point**	**Maximum Compressive strength (Mpa)**	
	**Vertical**	**Oblique**
a	434 ± 63^Aa^	267 ± 21^Ba^
b	208 ± 65^Ab^	286 ± 31^Aa^
c	134 ± 16^Ac^	95 ± 30^Ac^

^*^Same upper cases letters in row indicate no statistical significance between the vertically and obliquely groups within the each contact point (a,b and c)

^*^Same lower cases letters in column indicate no statistical significance among different contact points (a, b and c) within the vertically and obliquely embedded groups

## Discussion

This study employed multi directional loading conditions by embedding the specimens vertically and obliquely, while maintaining an identical occlusal design using three different inclinations at occlusal surface (Fig. 1). The occlusal surface design was set at 20 degrees for this study, however, in real clinical treatment, this inclination can be modified by decreasing it to avoid eccentric contacts or increasing it to facilitate occlusion.

In general, vertical loading conditions have demonstrated favorable outcomes compared to oblique loading conditions in dental biomechanics. A previous study conducted by Kim et al [14] assessed the fatigue behaviour of dental crowns subjected to single directional loading conditions and multi-directional loading conditions. In the latter, inclination angles were introduced in the facial-lingual and mesial-distal planes to simulate complex loading scenarios. The study concluded that multi-directional loading conditions could potentially replicate the vertical fracture pattern observed in real-world clinical situations.

In this study, different results were observed between different occlusal loading directions and contact points under the same load condition of 50N. In group V-a, the occlusal force is applied at the buccal cusp outer inclination of the implant (Fig. 9), the vector direction of the occlusal force according to the two-dimensional schematic is almost toward the center of the implant platform, this can be explained that the torque generation can be expected to be less. Likewise, group O-a, the vector direction is towards the center of the implant platform. However, angular deviation results show 0.06 for group V-a and 0.02 for group O-a with slight buccal tilting. This means that the generation amount of torque is small.

Removal torque evaluation results showed statistical differences, however, the results between contacts A, B and C were not predictable as for the angular deviation analysis. This can be explained by the screw loosening mechanism, which is caused by the restoration vibration due to an external force. Micromovement, fatigue characteristics of the screw and component misfit leads to screw loosening [15].

Goldberg et al. [16] conducted a study to assess the influence of abutment angulation on screw removal torque values under cyclic loading conditions. Three different inclinations were evaluated, 0 degrees, 20 degrees, and 28 degrees. The study findings revealed that the abutment angulation did not have a significant effect on the screw removal torque values. In the other hand, Hotinski and Dudley [17] reported that reducing the angle of the implant abutment can significantly enhance the resistance to screw loosening compared to straight implants.

However, screw loosening is multifactorial cause, and several parameters are associated [18]. Therefore, this type of vibration is not related to the vector direction. Nonetheless, there is a possibility that the tendency was not clear due to displacement of the experimental specimens caused by slippage, or any movement at the cyclic loading procedure. Furthermore, screw loosening is also associated to the bone condition which can be affected by high stress over the yield point of the bone [19].

For the fracture load results, the bending moment of the implant increases with the increase of the embedded angle. With-loading angles increase, the implant tends to fracture by a smaller number of cyclic-fatigue loading [20]. The results of this study showed similar results where the compressive strength resistance in vertically embedded group was higher compared to obliquely embedded group.

In this study, when the direction of occlusal loading is directed toward the center of the implant platform showed favorable results regarding to the angular deviation and maximum compressive strength. In the other hand, when the load is directed away from the center of the implant platform, the results were unfavorable. Thus, regardless of the same loading angle, the vector could be directed close to the center of the implant platform or away from according to the contact position (Fig. 9). Therefore, both loading angle and contact position should be considered simultaneously for predictable outcomes.

Apart from the restoration material, different loading direction and contact points might be an influencing factor in the fracture of the implant. This means that by considering the occlusal design to enhance force distribution, the compressive strength might obtain more favorable results compared when it is not accounted. This study evaluated not only the loading direction, but also different loading contact points were also assessed for the analysis

The present study was limited in that only one material (metal) was evaluated. Therefore, further investigations are necessary to evaluate the generalizability of our findings to different types of restorative materials. Incorporating various occlusal designs and conducting multi-directional analyses would also contribute to a more comprehensive understanding of the mechanical effects of implant-supported prostheses.

Furthermore, the use of finite element analysis would allow for a more detailed evaluation of stress distribution under different loading conditions analyzing specific areas of the prosthesis when the loading force is applied to identify potential failure mechanism. Overall, the findings of this study provide valuable insights into the mechanical behavior of implant-supported prostheses under different loading conditions. However, further investigation incorporating a wider range of materials and analytical techniques are necessary to fully understand the mechanical effects of implant-supported prostheses and optimize their design of clinical use.

## Conclusions

The direction of occlusal force is closely related to the location of loading contact points on restorations, and can significantly impact the distribution of stresses during masticatory function. It can therefore be inferred that the occlusal design of implant-supported prostheses, which incorporates appropriate occlusal force vectors and contact points, can enhance the predictability of definitive restorations.

## Data Availability

The authors declare that all data supporting the findings of this study are available within the article.

## Abbreviation

UTM: Univeral testing machine

## References

[1] Morton D, Gallucci G, Lin WS, Pjetursson B, Polido W, Roehling S (2018). Group 2 ITI consensus report: prosthodontics and implant dentistry. Clin Oral Implants Res.

[2] Lee H, Jo M, Noh G (2021). Biomechanical effects of dental implant diameter, connection type, and bone density on microgap formation and fatigue failure: a finite element analysis. Comput Methods Programs Biomed.

[3] Sugiura T, Yamamoto K, Horita S, Murakami K, Tsutsumi S, Kirita T (2017). Effects of implant tilting and the loading direction on the displacement and micromotion of immediately loaded implants: an in vitro experiment and finite element analysis. J Periodontal Implant Sci.

[4] Hosseini-Faradonbeh SA, Katoozian HR (2022). Biomechanical evaluations of the long-term stability of dental implant using finite element modeling method: a systematic review. J Adv Prosthodont.

[5] Javed F, Romanos GE (2010). The role of primary stability for successful immediate loading of dental implants. A literature review J Dent.

[6] Holmgren EP, Seckinger RJ, Kilgren LM, Mante F (1998). Evaluating parameters of osseointegrated dental implants using finite element analysis–a two-dimensional comparative study examining the effects of implant diameter, implant shape, and load direction. J Oral Implantol.

[7] Kapoor S, Rodrigues S, Mahesh M, Shetty T, Pai U, Saldanha S (2021). Evaluation of stress generated with different abutment materials and angulations under axial and oblique loading in the anterior maxilla: Three-dimensional finite element analysis. Int J Dent.

[8] Tak S, Noh G, Jeong Y, Lee H (2021). Automated vector analysis to design implant-supported prostheses: a dental technique. J Prosthet Dent.

[9] Duyck J, Van Oosterwyck H, Vander Sloten J, De Cooman M, Puers R, Naert I (2000). Magnitude and distribution of occlusal forces on oral implants supporting fixed prostheses: an in vivo study. Clin Oral Implant Res.

[10] Binon PP, McHugh MJ (1996). The effect of eliminating implant/abutment rotational misfit on screw joint stability. Int J Prosthodont.

[11] Setia G, Yousef H, Ehrenberg D, Luke A, Weiner S (2013). The effects of loading on the preload and dimensions of the abutment screw for a 3-unit cantilever-fixed prosthesis design. Implant Dent.

[12] Sammour SR, Maamoun El-Sheikh M, Aly El-Gendy A (2019). Effect of implant abutment connection designs, and implant diameters on screw loosening before and after cyclic loading: In-vitro study. Dent Mater.

[13] Huang Y, Wang J (2019). Mechanism of and factors associated with the loosening of the implant abutment screw: a review. J Esthet Restor Dent.

[14] Kim WH, Song ES, Ju KW, Lim D, Han DW, Jung TG, Jeong YH, Lee JH, Kim B (2020). Mechanical assessment of fatigue characteristics between single- and multi-directional cyclic loading modes on a dental implant system. Materials (Basel).

[15] Lee J, Kim Y-S, Kim C-W, Han J-S (2002). Wave analysis of implant screw loosening using an air cylindrical cyclic loading device. J Prosthet Dent.

[16] Goldberg J, Lee T, Phark JH, Chee W (2019). Removal torque and force to failure of non-axially tightened implant abutment screws. J Prosthet Dent.

[17] Hotinski E, Dudley J (2019). Abutment screw loosening in angulation-correcting implants: an in vitro study. J Prosthet Dent.

[18] Kourtis S, Damanaki M, Kaitatzidou S, Kaitatzidou A, Roussou V (2017). Loosening of the fixing screw in single implant crowns: predisposing factors, prevention and treatment options. J Esthet Restor Dent.

[19] Feng X, Lin G, Fang CX, Lu WW, Chen B, Leung FKL (2019). Bone resorption triggered by high radial stress: the mechanism of screw loosening in plate fixation of long bone fractures. J Orthop Res.

[20] Suzuki H, Hata Y, Watanabe F (2016). Implant fracture under dynamic fatigue loading: influence of embedded angle and depth of implant. Odontology.

